# Characterization and Comparison of the Rumen Luminal and Epithelial Microbiome Profiles Using Metagenomic Sequencing Technique

**DOI:** 10.3389/fvets.2022.799063

**Published:** 2022-02-24

**Authors:** Ricardo M. Stockler, Haley Hallowell, Keah V. Higgins, Erin S. Groover, Elizabeth M. Hiltbold, Benjamin Newcomer, Paul H. Walz

**Affiliations:** ^1^Department of Clinical Sciences, College of Veterinary Medicine, Auburn University, Auburn, AL, United States; ^2^Department of Biological Sciences, College of Sciences and Mathematics at Auburn University, Auburn, AL, United States; ^3^Veterinary Education, Research, and Outreach Program, Texas A&M and West Texas A&M Universities, Canyon, TX, United States; ^4^Department of Pathobiology, College of Veterinary Medicine, Auburn University, Auburn, AL, United States

**Keywords:** rumen cannulation, *in vivo* microbiome, bovine microbiome, metagenomic analysis, bioinformatics

## Abstract

Bacterial dysbiosis as a result of nutritional, bacterial, viral, and parasitic gastrointestinal infections can adversely affect the metabolism, productivity, and overall health of cattle. The purpose of this project was to characterize the commensal microbiota present in two locations of the rumen concomitantly *in vivo* with the animals undergoing habitual husbandry, as it was hypothesized that there are major differences in the commensal microbiota present in the two locations of the adult bovine major forestomach. A surgically fitted rumen cannula was used to allow ruminal lumen contents and mucosal biopsies to be collected from six crossbred yearling steers. In order to assess as much environmental and individual steer microbiota variation as possible, each animal was randomly sampled three times over a 3 week period. 16S rRNA sequencing was performed to provide a detailed descriptive analysis from phylum to genus taxonomic level. Significant differences were observed between luminal and epimural bacterial populations in the bovine rumen. As expected, a core microbiome composed by *Firmicutes* and *Bacteroidetes* represented over 90% of the microbiome, however, further analysis showed distinct diversity and distribution of the microbiome between the two locations. Characterizing the gastrointestinal microbiome *in vivo* is imperative. The novelty and the contribution of this study to the literature is the use of live cattle which allowed real-time sample collections and analysis of the rumen microbiome providing an understanding of what is normal in the live animal.

## Introduction

The ruminal bacterial population plays an important role in the dietary metabolism of the host, including nutrient consumption and utilization, and consequently, manipulation of the rumen microbiota is known to affect animal performance, production, sustainability and ultimately profitability (1). The commensal microbiome plays an important role in nutrient and energy extraction and energy regulation (2). In ruminants, specifically cattle, the composition of the rumen microbiome and its impact on health, nutrition, and host physiological parameters have been studied (3–12).

Knowledge gaps are present with respect to the rumen epithelium and its unique interaction between host and microbial metabolism. Biopsy sampling techniques of the rumen epithelium have been used to analyze the effects of dietary transition on ruminal epithelial gene expression and the effects of diet on rumen epithelial development (13, 14). However, full understanding of the true commensal microbiome in cattle is still limited, especially with respect to what ensues at the epithelial surface of the rumen when cattle are undergoing normal husbandry.

The 16S rRNA gene sequencing technique is a more commonly used strategy to study the rumen microbiome. Multiple publications can be found that use this method to study the microbiome; however, methodology and analysis of the taxonomic data collected are still known difficulties encountered by microbiome researchers using this method (1).

The commensal microbiota composition of the rumen is largely determined by dietary factors. However, age, breed, and species are also known factors that impact rumen health (15, 16). Enzymes necessary for digestion via fermentation of the diet consumed by ruminants are provided by the commensal rumen microbiome. Also, the microbiota is responsible for the synthesis of many amino acids and vitamins that are later absorbed in the small intestine to fulfill the host requirements (17).

The characterization of the ruminal and fecal microbiome and its impact on bovine health, production has been investigated (7, 9, 18). In those studies, milk yield and composition were found to be highly correlated with the abundance of various bacterial members of the rumen microbiome, specifically the impact between the *Firmicutes*:*Bacteroidetes* ratio (F:B ratio) on milk-fat yield (9). A later study investigated the composition of bacterial microbiota in the rumen content, epithelium, and feces of dairy cattle. The study demonstrated remarkable compositional differences among the three locations, suggesting that bacterial communities are specific and adapted to their specific microenvironment (18).

At the phylum level, *Bacteroidetes* and *Firmicutes* are among the primary metabolically-active bacteria with a critical role in breaking down plant wall compounds and host-derived carbohydrates, including particles attached to the mucins or chondroitin sulfates of the protective mucosal layer of the intestine (2). The F:B ratio has been demonstrated to affect energy dietary uptake and expenditure leading to obesity in pigs, mice and humans (2, 19).

The rumen microbiome profile is dependent on the composition of substrate that has been offered, such as the proportions of cellulose, hemicellulose, pectin, starch, and amino acids. Further into the taxonomic analysis, it is reported that *Butyrivibrio fibrisolvens, Prevotella ruminocola, Ruminococcus flavefaciens*, and *Ruminococcus albus* are known to be responsible for the digestion of hemicellulose and cellulose rich diets, such as those composed primarily of forages (20). The digestion of grain-based (high starch) diets is accomplished by *B. fibrisolvens, Prevotella ruminocola, Fibrobacterer succinogenes, Clostridium species, Streptococcus bovis, Ruminobacter amylophilus, Succinimonas amylolytica*, and *Selenomonas ruminantium*, in addition, amino acids are readily fermented by bacteria belonging to the genus *Prevotella* to produce adenosine triphosphate (ATP) (20).

The aim of this project was to characterize and describe the gastrointestinal (GIT) commensal microbiome present in the lumen and the epimural surface (microbiota that is firmly attached to the rumen wall) of the rumen of cattle undergoing normal husbandry. It was hypothesized that due to metabolic processes and/or host properties, there are differences in the natural microbiota present in the epimural surface and luminal contents of the adult bovine major forestomach.

## Materials and Methods

### Animals

The study was conducted in mid spring in the southeast region of the USA at Auburn University College of Veterinary Medicine, following approval of all procedures by the campus Institutional Animal Care and Use Committee (PRN 2015-2676). Six dairy crossbred steers weighing an average of 249 kg (240–277 kg; 530–610 lbs.) were used in this study. The cattle were housed in grass pasture, fed one flake of Bermuda grass hay and five pounds of soy hull pellets per head twice daily, and offered water *ad libitum*. All steers were surgically fitted with a three-inch rumen cannula^1^. The surgical procedure was performed as described by Laflin and Gnad (21). Post-operative treatment consisted of 2.2 mg/kg of ceftiofur hydrochloride^2^. administered subcutaneously once daily for 5 days and 1.0 mg/kg of meloxicam^3^ administered orally once daily for 5 days in addition to daily cleaning of the inserted cannula. A 3-month recovery period was observed following surgery to allow for complete healing of the surgical sites, ensure appropriate drug withdrawal periods were met, and provide research animals a consistent diet prior to study initiation and sample collection. Once the recovery period elapsed, the cattle were housed in the same pasture throughout the length of the study without fence-to-fence contact with other animals, and were consistently fed five pounds of a 50:50 mixture soy hull and corn gluten pellets plus one flake of Bermuda grass hay (~3 pounds) per head per day. To ensure consistency and eliminate dietary bias, this nutritional scheme remained the same throughout the study collection period.

### Study Timeline and Sample Collection

In order to optimize consistency and still assess potential variation due to individual, environmental, and bacterial factors, a simple randomization technique using the flip of a coin was used to assign each animal randomly weekly over a 3-week period (Table 1).

**Table 1:** Timeline for sample collection.

	**Week 1**	**Week 2**	**Week 3**
Monday			50, 69, 70
Tuesday		93, 10, 50	
Wednesday	71, 70, 93		93, 10, 71
Thursday		70, 69, 71	
Friday	10, 69, 50		

*Chart numbers refer to individual steer numbers used in the study*.

**Figure 1:**
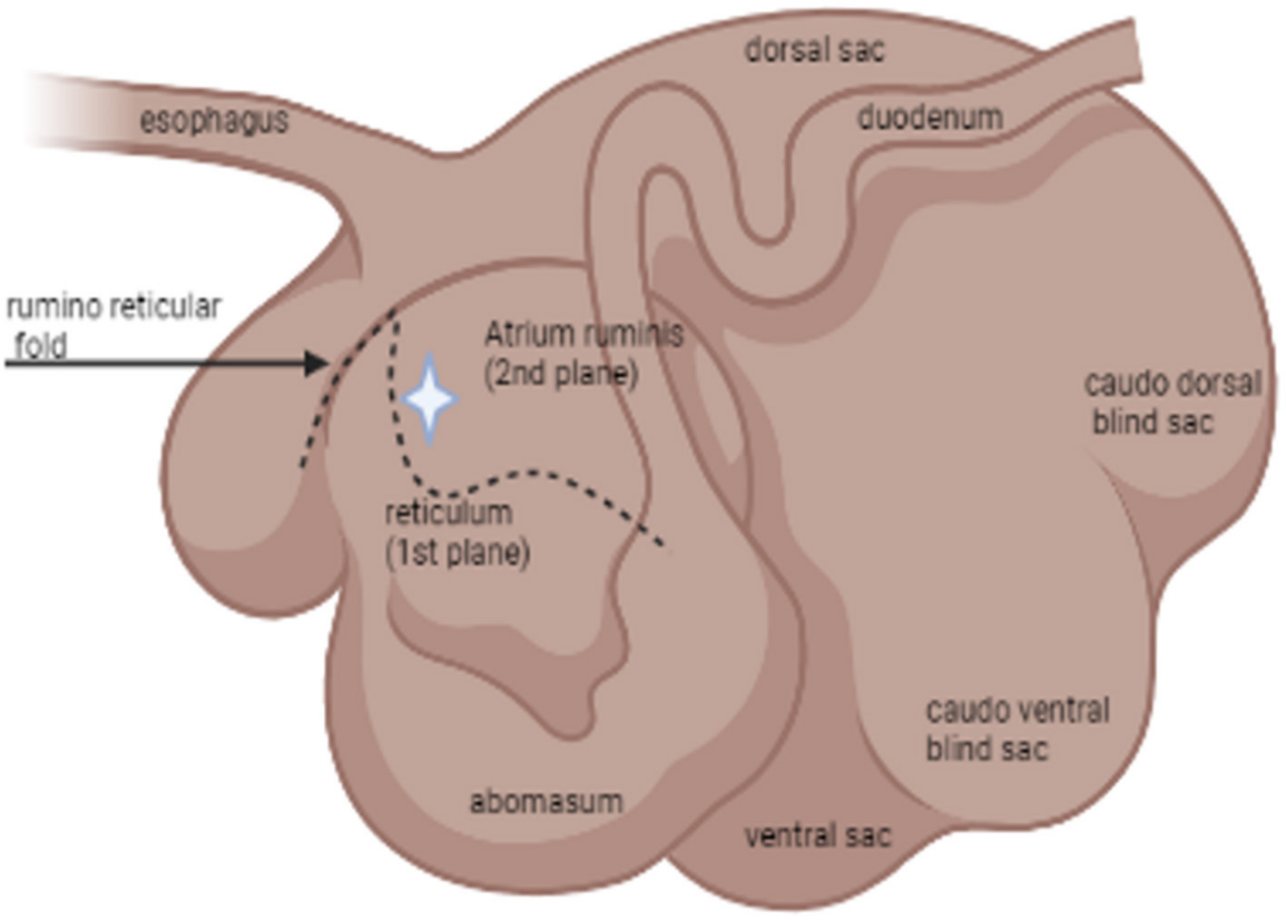
Schematic illustration by BioRender^®^ of the relevant anatomy of the rumen: lateral view. The star icon indicates the location of where samples were collected *via* rumen cannula.

For sample collection, each individual animal was haltered and restrained in a livestock chute system. The rumen cannula was opened manually. Using a sterile double-gloved sleeve, the sample collector entered the rumen and manually palpated the atrium ruminis and the ruminoreticular fold (Figure 1). A sample of the ingesta from the ventral aspect of the atrium ruminis (located caudally to the ruminoreticular fold) was collected using a snap cap collection vial. After collection, the cap was closed inside the atrium ruminis of the rumen before removal to minimize potential contamination of samples. Such samples were designated as lumen contents. Next, epithelial biopsy samples were collected from the atrium ruminis of the rumen using a 54 cm Jackson uterine biopsy forceps (Jorgensen Labs INC.). Using a new sterile double-gloved sleeve, the sample collector entered the rumen with the forceps covered by a sterile sleeve. Once the atrium ruminis was located and the biopsy site identified (Figure 1, star icon), the “push through” technique was used to expose the forceps allowing the biopsy of the rumen epithelium to be taken. The forceps were pulled back in the sleeve before removal from the rumen by the sample collector. All samples were placed in 750 μl of RNAlater immediately after collection and stored at 4°C until processed.

### Sample Processing

#### DNA Isolation

A total of 18 luminal samples and 18 mucosal biopsy samples were collected for analysis and subsequent sequencing. Isolation of DNA from all samples was extracted using a commercial kit^4^ according to the manufacturer‘s guidelines for DNA extraction in tissue, using glass beads, and for fluid samples. The pathogen detection protocol allows rapid and reliable isolation of purified DNA using a combination of reversible nucleic acid-binding properties of ^5^HiBind^®^ matrix and spin column technology to allow the elimination of humic acid, polysaccharides, phenolic compounds, and enzyme inhibitors. The extracted DNA was eluted into 100 μl of sterile elution buffer and stored at −20°C until the time of DNA sequencing and bioinformatics analysis.

### 16S rDNA Sequencing and Bioinformatic Analysis

The bacterial microbiome was analyzed using 16S rRNA gene V4 variable region PCR primers 515/806 in a single-step 30 cycle PCR using a commercially available kit following a published protocol (22). Sequencing was performed on an Ion Torrent PGM (Personal Genome Machine) following the manufacturer's guidelines and processed using a proprietary analysis pipeline at^6^ MR DNA laboratory.

Sequences were de-multiplexed and sequence adaptors were removed prior to QIIME analysis (23). Bacterial composition was assessed using the Quantitative Insights into Microbial Ecology (QIIME) suite, QIIME2 version 2019. Reads were filtered for length and quality and chimeras were removed. Sequences were clustered into operational taxonomic units (OTUs) with a 97% identity threshold. Taxonomic assignment was performed using BLASTn classifier (trained by the SILVA database, release version 132) (24). OTUs with an abundance below 20 and present in less than five samples were not included in the downstream analysis. Remaining OTUs were consolidated into an OTU network for all individual samples using QIIME2 and this was imported into RStudio for downstream analysis.

### Data and Statistical Analysis

Individual samples from each group were used to assess microbial abundance and variation for both sampling strategies. Alpha diversity was assessed through rarefaction graphs constructed with QIIME2. Relative abundance was used to calculate means and standard deviations of each group at each time point using the statistical program R (25). Using the RStudio statistical platform, *t*-tests were performed to identify significant difference in relative abundance of microbial taxa. Non-metric multidimensional scaling (nMDS) ordination was generated in RStudio using the *vegan* package (26). To generate the nMDS, raw bacterial hits were used to compute a sample dissimilarity matrix using the Bray-Curtis dissimilarity index. This matrix was then used to compute an ordination of the samples in two dimensions. The *vegan* package was also used to calculate Shannon's Diversity Index scores. Then, the Pielou's Evenness Index was calculated by dividing the Shannon's Diversity Index score by the log of unique species amount. Lastly, the microbial community structure was analyzed using weighted UniFrac distance matrices. Principal coordinate analysis plots were used to visualize the data in these matrices, and pairwise analysis of similarities (ANOSIM) was utilized to determine if there were any significant differences between the microbial communities. Significance reported for any analysis was defined as *p* < 0.05.

## Results

After stringent quality sequence curation, a total of 2,239,622 sequences were parsed and 2,074,523 were then clustered. A total of 2,071,427 sequences identified within the bacteria domains were utilized for final microbiota analyses. The average reads per sample was 51,785.

**Figure 2:**
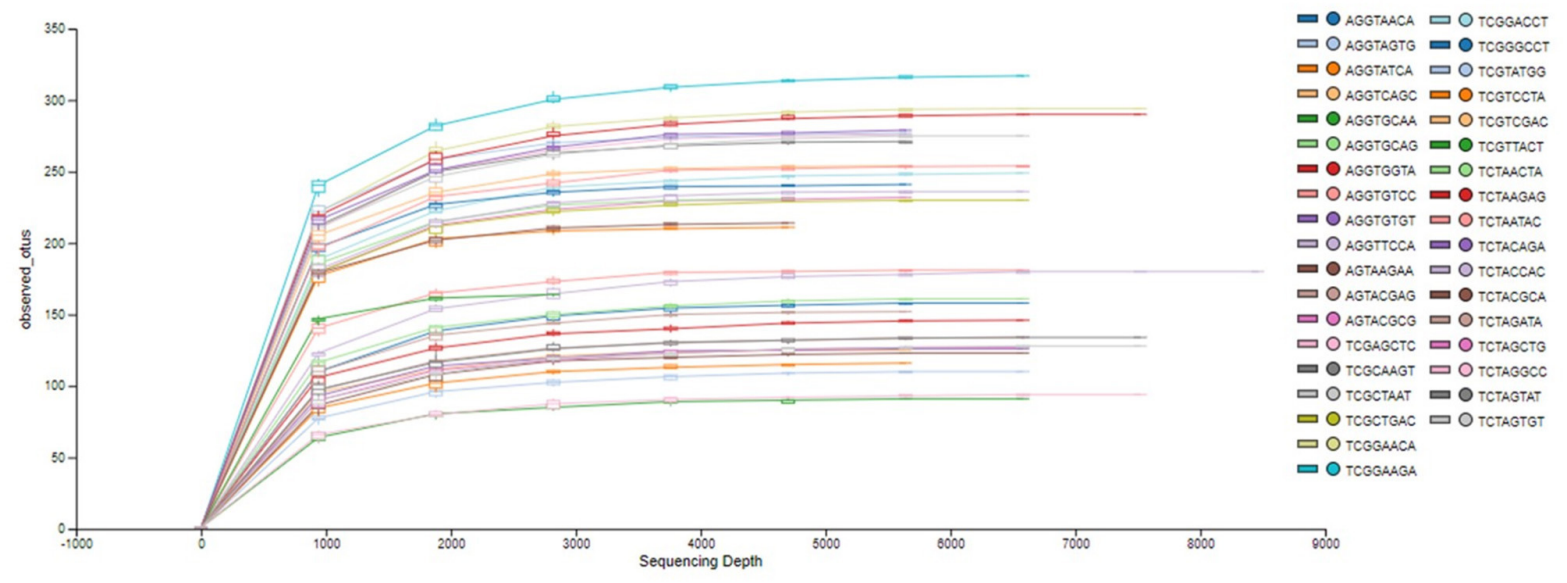
Phylogenetic rarefaction curves estimating species richness. The rarefaction curves produce smoother lines facilitating full dataset comparison by reaching a clear asymptote.

The analysis of the bacterial diversity is a function of sequencing effort and represented as individual samples by the color-coded lines. The positive assessment of richness for each sample collected is determined by the fact that each color-coded line achieved its maximum peak and plateau consistently with each other signifying adequate depth of sampling and alpha diversity (Figure 2).

**Figure 3:**
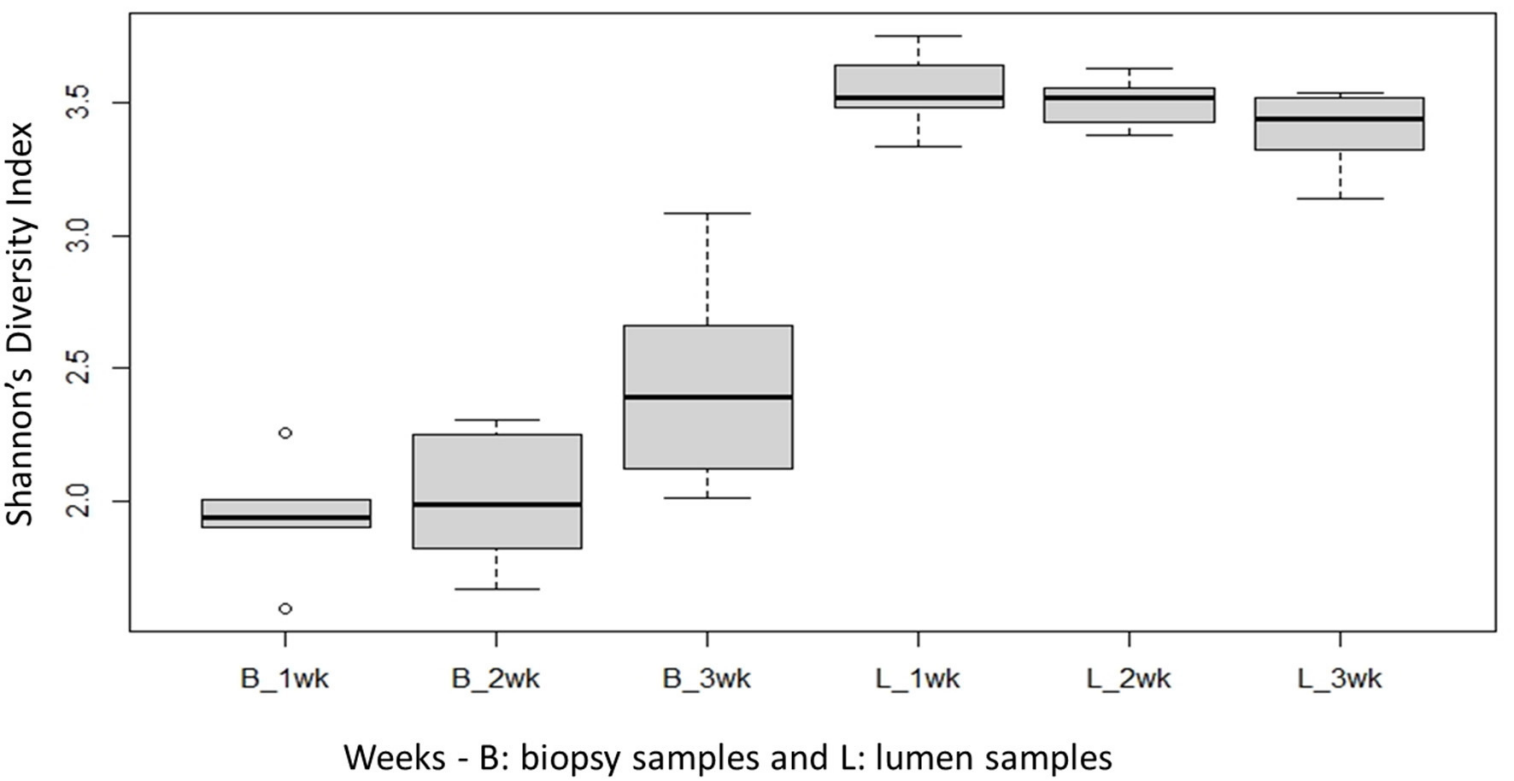
Comparison of the average bacterial OTU‘s Shannon index diversity for the mucosal surface (B-biopsy) and lumen contents (L, lumen samples) for each week sampled.

**Figure 4:**
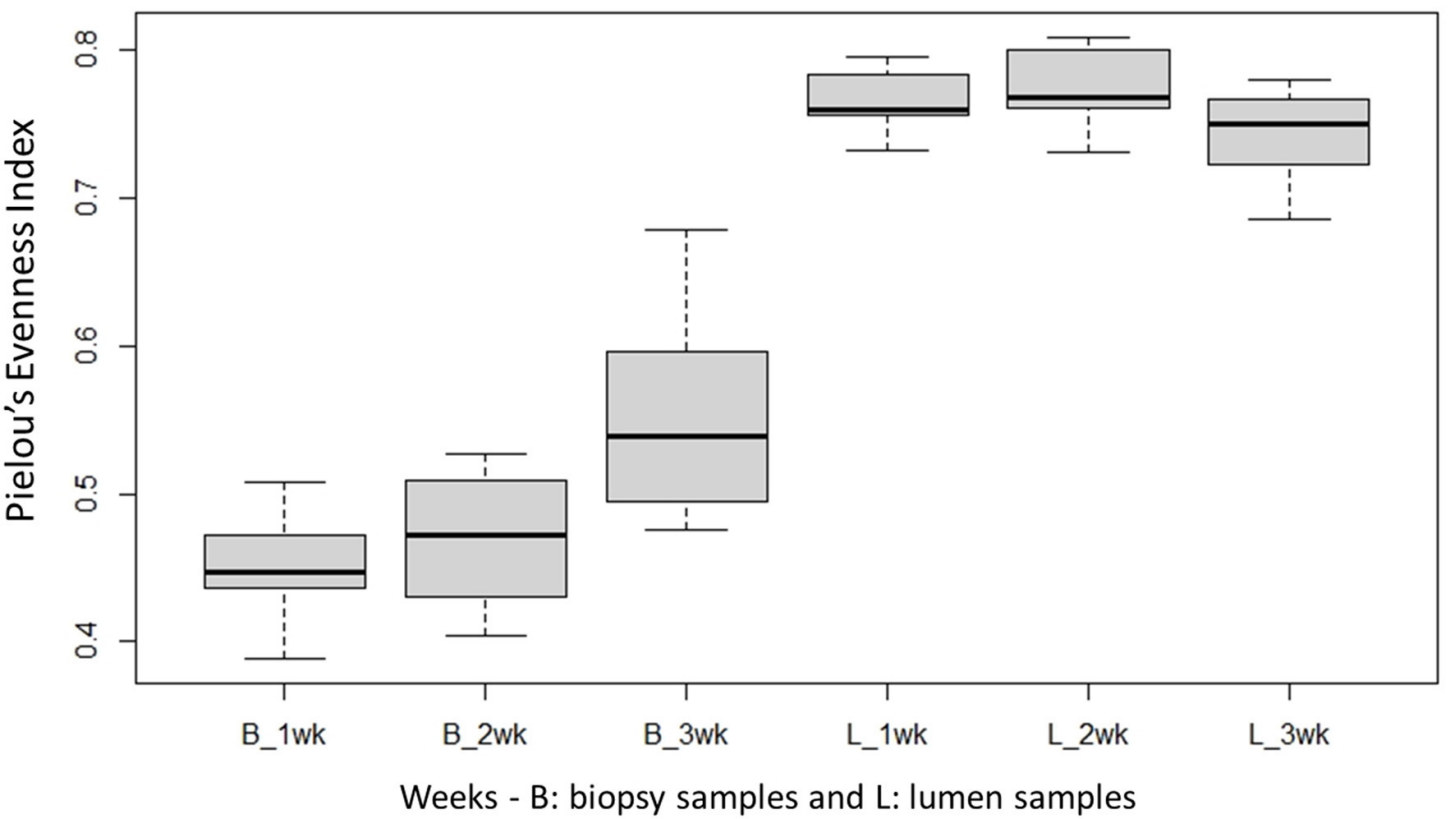
Comparison of the average bacterial OTU‘s Pielou's evenness index for the mucosal surface (B, biopsy) and lumen contents (L, lumen samples) for each week sampled.

Species richness between the two locations, mucosal surface and lumen contents, was measured using the Shannon-Wiener Index (Figure 3), while evenness was measured utilizing Pielou's Evenness Index (Figure 4). Throughout the experiment, minimal change was observed in the diversity and evenness within the microbiota, however for the lumen samples there was a trend that for increasing in the diversity and evenness regardless of time. This is confirmed by the lack of statistical significance of the Shannon index reporting a *p*-value = 0.40 for the mucosal surface samples and 0.44 for the lumen contents, and for the evenness trend at 0.40 and 0.36 for the mucosal surface and the lumen contents, respectively.

**Figure 5:**
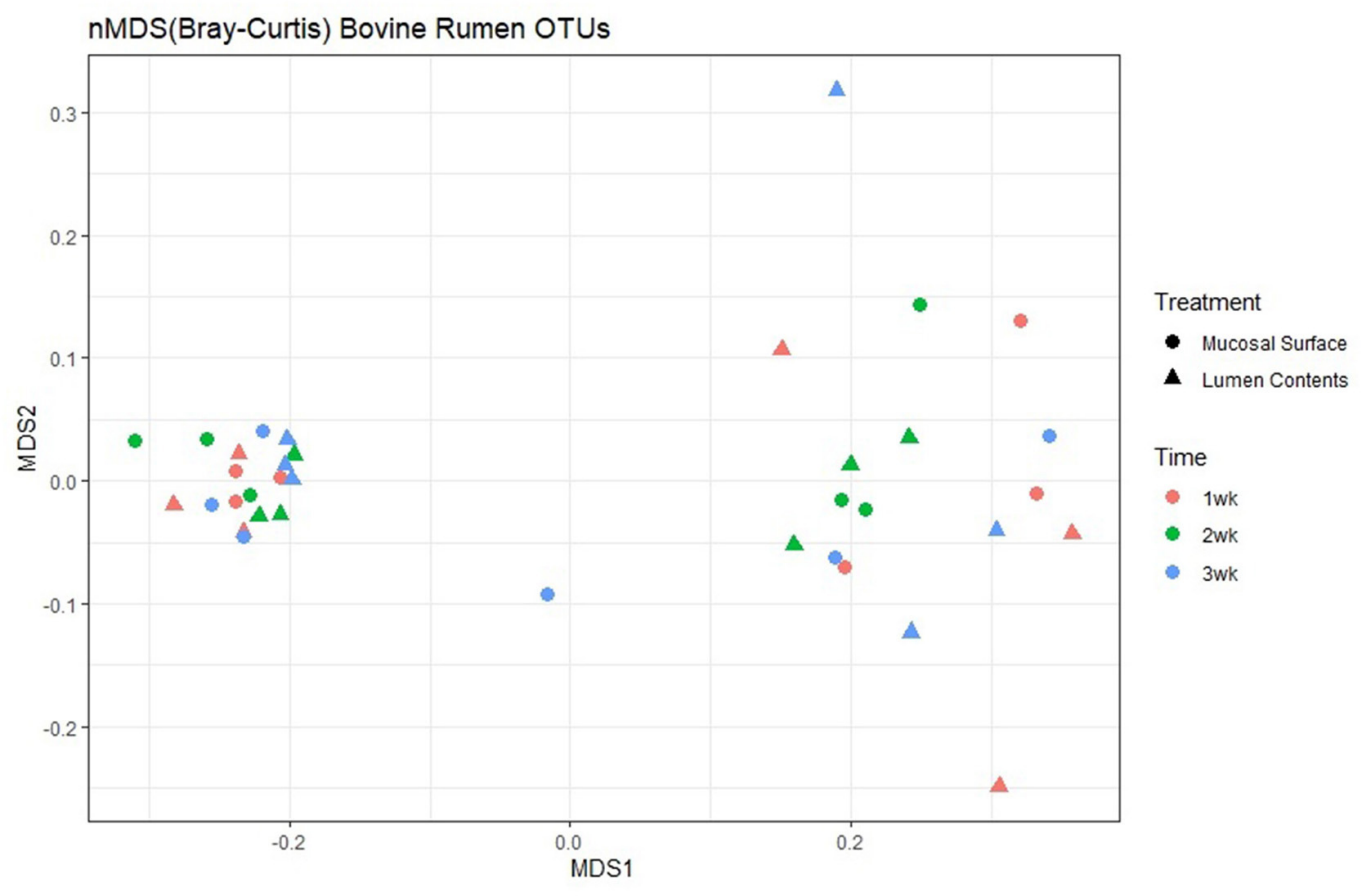
Multidimensional scaling plot (MDS) of bacterial lineages in the mucosal surface (biopsy) and lumen contents by time.

Next, to determine the amount of dissimilarity seen in the microbiota associated with the lumen and mucosal surface, an nMDS ordination plot utilizing a Bray-Curtis dissimilarity index was generated and valued at 0.112 (Figure 5). Figure 5 demonstrates a distinct separation of samples in the ordination plot, suggesting the microbiota between the two locations are dissimilar to each other regardless of timepoint as displayed by two distinct clusters of the same samples. As stated above, the stress index statistic calculated indicated a fair to good fit of the data to the model utilized.

**Figure 6:**
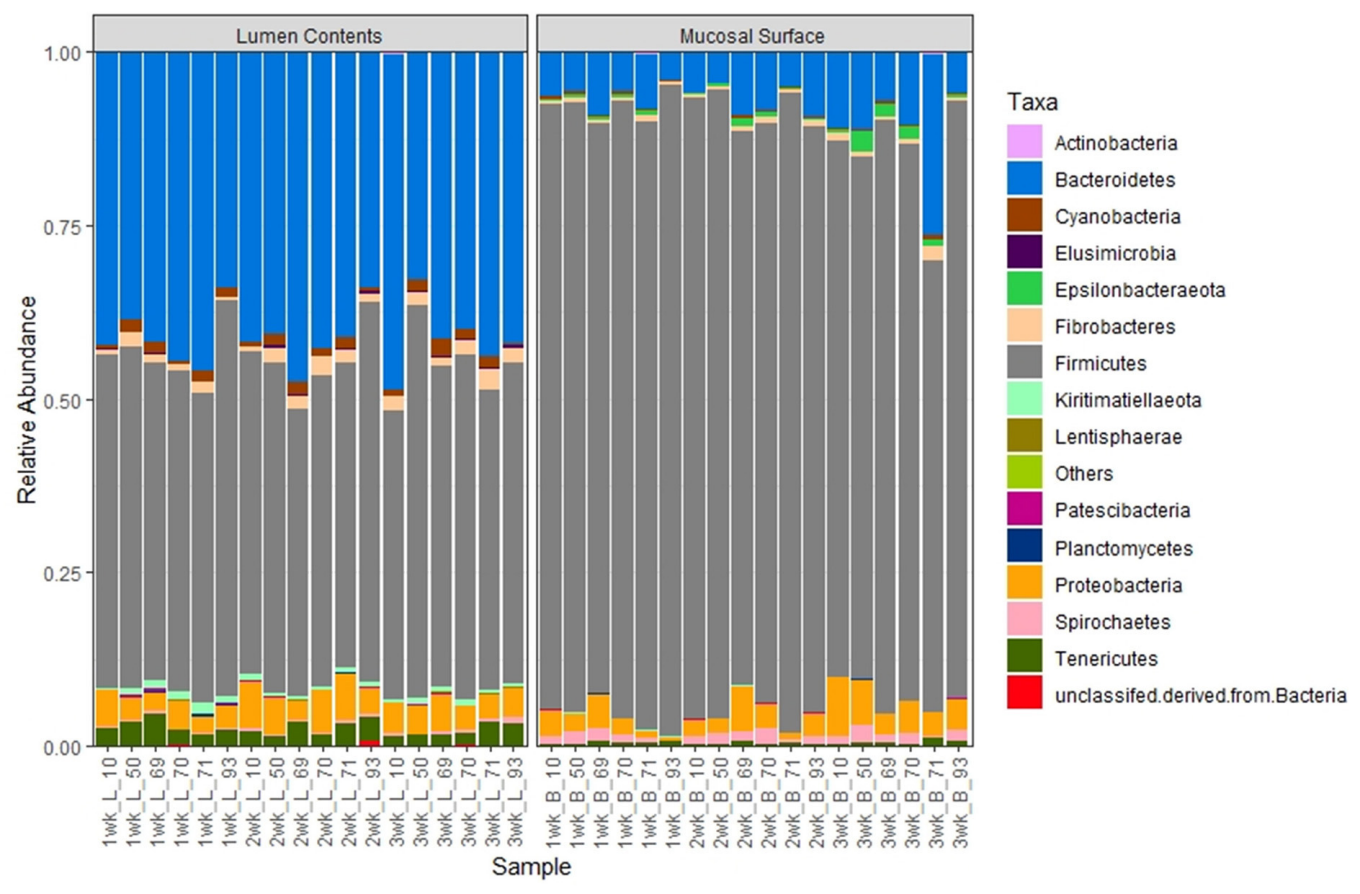
Bacterial phylum. Stacked bar chart representing the abundance of the microbiota at the phylum taxonomic level in the mucosal biopsy surface and lumen contents across the sampled weeks for each animal.

At the phylum level, Firmicutes (86.6%) and Bacteroidetes (6.2%) followed by smaller percentages of Proteobacteria (3.7%) and Spirochetes (1%). were the most abundant bacteria in the epimural biopsy samples. In contrast, Firmicutes (55.3%), Bacteroidetes (30.7%), Proteobacteria (6.7%), Fibrobacteres (1.3%) and Tenericutes (1.3%) were the most abundant bacteria present in the luminal contents. Although no statistical significance was annotated, (*p* = 0.65, overall) the F:B ratio in the mucosal biopsy samples appeared to be numerically higher relative to the samples collected from the luminal contents (Figure 6).

**Figure 7:**
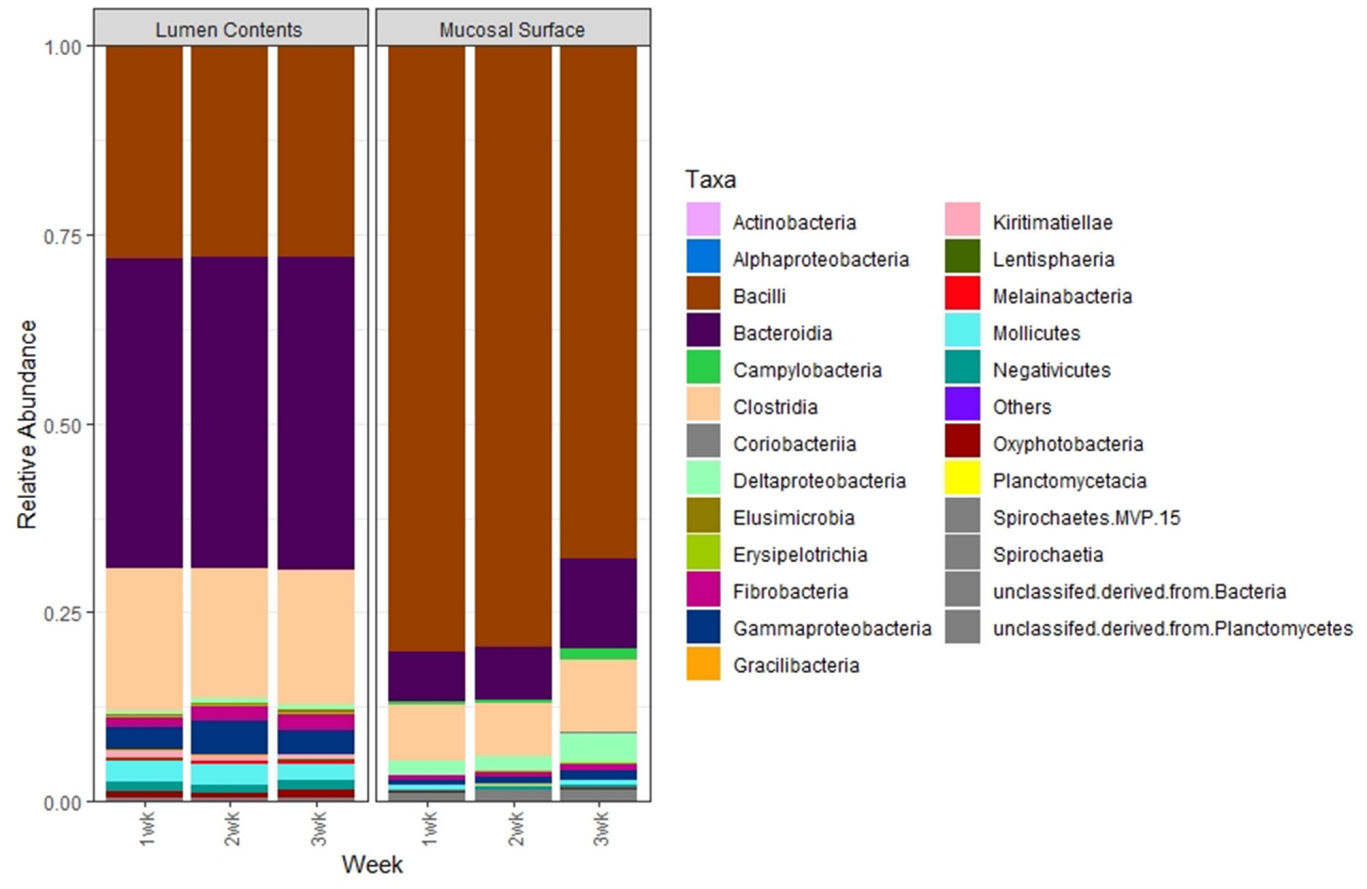
Bacterial class. Stacked bar chart representing the average abundance of the microbiota at the class taxonomic level in the mucosal biopsy surface and lumen contents across the sampled weeks.

To further study the distribution of these two predominant phyla at a lower taxonomic level, we investigated the distribution of microbial populations at the Class, Order, Family and Genus levels for the most abundant phyla. At the Class level, in the lumen contents, the vast majority of the total Firmicutes (55%) in the lumen contents were represented by Bacilli (30.5%) and Clostridia (20.9%) constituting over 90%, while in the epimural surface, Bacilli (76.5%) and Clostridia (9.1%) were the top classes represented (>95%) within that phylum. Conversely, minimal variation was observed for the Bacteroidetes phylum regardless of the location of the sample, with over 25% represented by Bacteroidia in the lumen vs. 5% in the biopsy (Figure 7). Similarly, even though the absence of statistical significance was noted, *p* = 0.63, when the OTU abundance between the two locations was compared, numerically the differences were evident.

**Figure 8:**
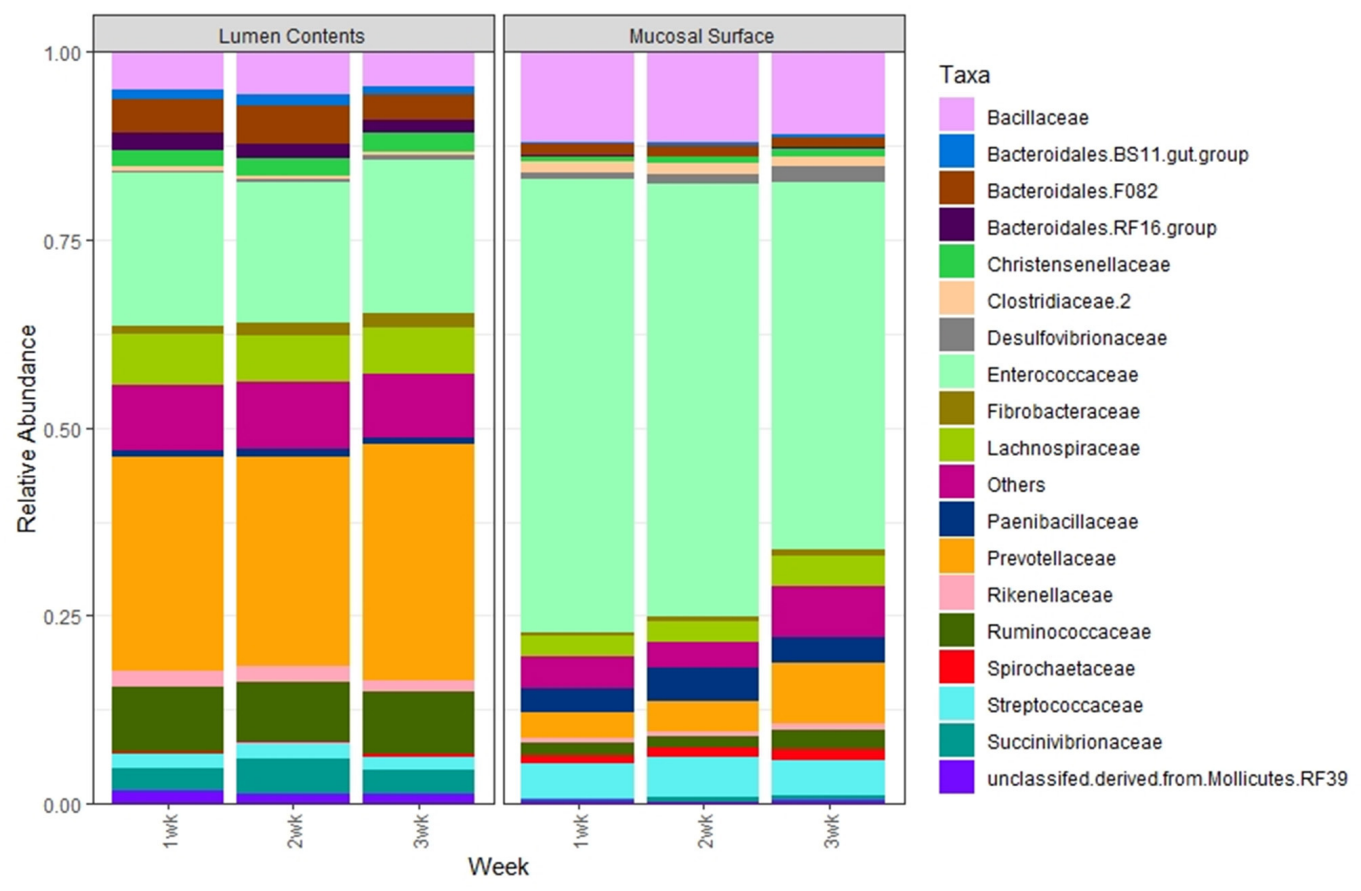
Bacterial family. Stacked bar chart representing the average abundance of the microbiota at the family taxonomic level in the mucosal biopsy surface and lumen contents across the sampled weeks.

**Figure 9:**
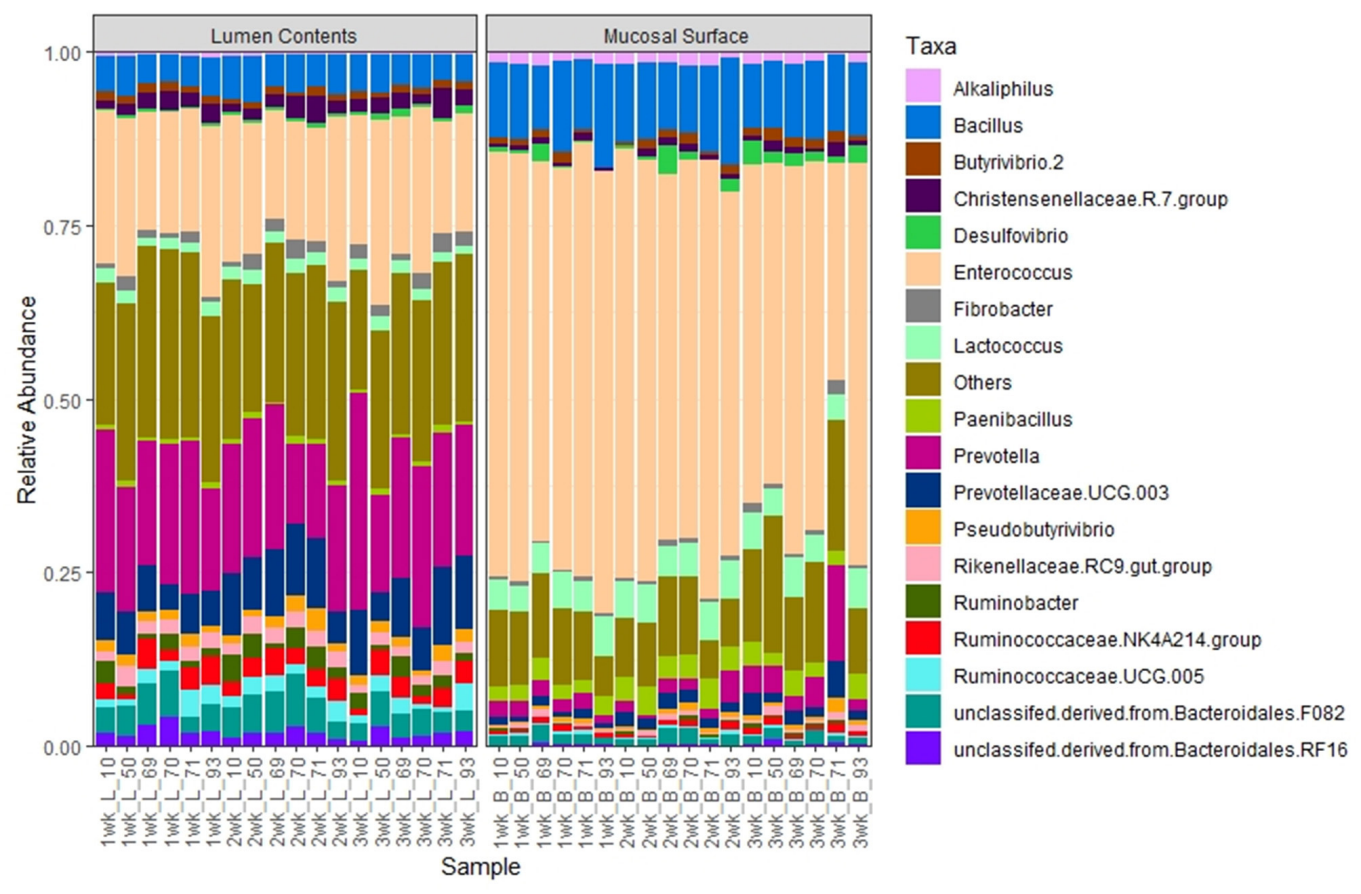
Bacterial genus. Stacked bar chart representing the abundance of the microbiota at the genus taxonomic level in the mucosal biopsy surface and lumen contents across the sampled weeks for each animal.

The same abundance pattern was observed at the Family and Genus levels with the Enterococcaceae and Enterococcus (*p* < 0.01) representing over 25% in the lumen samples vs. over 65% in the epimural surface, whereas the Prevotellaceae and Genus *Prevotella* (*p* < 0.01) is present in over 14% of the samples associated with the lumen in only a little over 2% of samples associated with the epimural surface of the rumen (Figures 8, 9).

**Figure 10:**
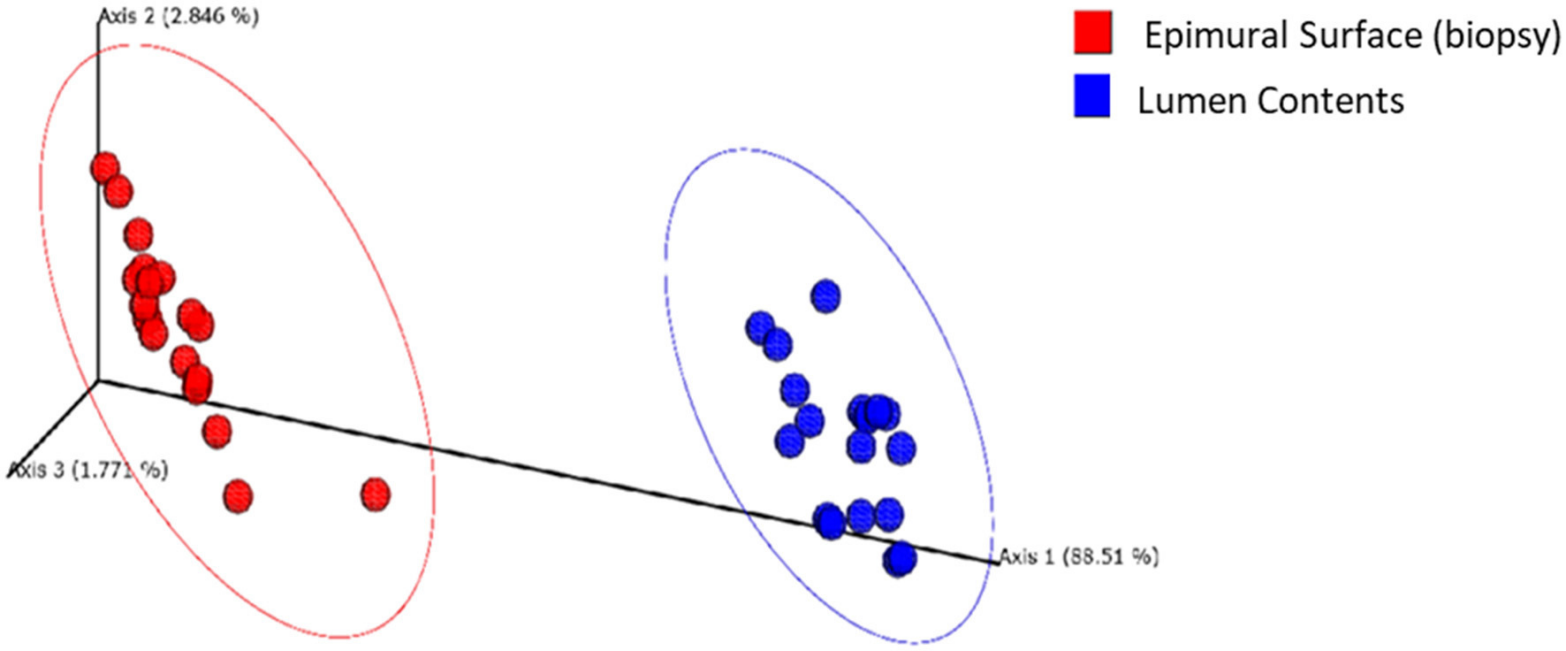
Principal coordinate plot of weighted UniFrac data.

Phylogenetic assemblage amongst the epimural surface sample was significantly different (*p* = 0.001) from the lumen contents samples. Primary vector explains 88.5% of the variation between the groups. The first 3 vectors together exhibit 93.1% of the variation among the groups, *p* = 0.001 (Figure 10). Based on the ANOSIM R value in Table 2, we can confidently indicate the most similar samples are in the same group (*R* = 0.99).

**Table 2:** Pairwise ANOSIM of weighted UniFrac distance matrix.

**Group 1**	**Group 2**	**Sample size**	***R***	***p*-value**
Epimural surface	Lumen contents	36	0.999	0.001

## Discussion

In this study, numerically significant differences obtained via target gene sequencing were demonstrated between luminal and epimural bacterial populations in the bovine rumen. Although the rumen microbiome has been investigated using different methods, the novelty of this study is the characterization of the microbiota present in two locations of the rumen concomitantly *in vivo* with the animals studied were undergoing customary husbandry.

The maintenance of healthy and stable ruminal fermentation is known to be critical for ruminants to preserve their rumen bacterial populations and functional fermentation and digestion. Metabolism of nutrients is key in the symbiotic relationship between the host and microbiome. Significant differences obtained via 16S rRNA sequencing were observed between luminal and epimural bacterial populations in the bovine rumen. The higher species abundance observed for the epimural communities suggests their core importance metabolically and immunologically to the host. These findings are in agreement with a study published by a group from Canada that suggested that the core metabolically active epimural bacterial population can survive mucosal immune defense mechanisms and may be crucial for priming the host mucosal immune system (27).

Using cultivation-based analysis, Creevey et al. (3), reported the existence of nine phyla in the rumen, with *Firmicutes, Proteobacteria* and *Actinobacteria* comprising 90% of the cultures. Similarly, the top 3 phyla reported in this manuscript associated with the luminal samples were *Firmicutes* (55.3%), *Bacteroidetes* (30.7%) and *Proteobacteria* (6.7%) which made up the top 90%, whereas, Firmicutes alone composed over 85% of the microbiota present on the epimural surface (3). The variation of lesser abundant bacteria, beyond the anticipated core microbiome, is speculated to be related to the dietary uniqueness of the individual. In addition, the significant abundance of *Firmicutes* at the phylum level and *Bacilli* (~75%) at the Class taxonomic level found on the epimural surface was expected as those bacteria play an active role in ruminants with respect to carbohydrate metabolism (28).

Previous research suggested the existence of a core microbiome in the bovine rumen, and even though variability was great, the authors demonstrated a high phylogenetic correlation among the described genera (3, 7, 8). In another study, the same researchers examined the rumen microbiome in lactating cows (8). The results were consistent with those of the first study also in that both studies demonstrated the presence of a core microbiome in the rumen (7, 8). Specifically, they reported a bacterial population with 32% of the operational taxonomic units (OTUs) shared by at least 90% of the animals in the study and 19% of the OTUs common to 100% of the animals. Similar to previous studies, the samples evaluated in the current study over a 3-week period demonstrated constant taxonomic characteristics, also representing a core rumen microbiome with minimal variation between animals and weeks, however, with notable significant variation existing between locations, specifically in reference to the *Prevotella* and *Enterococcus*.

The importance of the F:B ratio has been analyzed in mice and human studies, where imbalances in the ratio in the GIT has been demonstrated to affect obesity and the capability of the host to harvest energy (2, 19). The microbiome present in obese hosts demonstrated greater capacity to harvest energy from the diet. Therefore, obesity in the host was supported and even exacerbated by the imbalanced bacterial populations (29, 30). In this study, consistency in the ratio throughout the project between the two locations was observed. The authors speculate this result was due to using healthy subjects under habitual and consistent husbandry during the study period.

A positive relationship between the rumen microbiome and certain physiological parameters in the lactating dairy cow has been identified (9). The group reported a strong correlation between milk fat yield and the F:B present in the ruminal contents. The specific presence of *Prevotella* bacteria, up to 72% of the bacterial population in some samples, negatively affected milk fat yield in those cattle (9). Results of the current study demonstrated that *Prevotella* was found in significantly greater abundance in the lumen samples compared to the epimural surface, approximately 14 vs. 2%, respectively. This finding is consistent with the fact that *Prevotella* in the rumen is physiologically responsible for the prevention of the colonization of acid-producing bacteria which are known to disrupt the overall digestive processes in ruminants. This finding likely explains the higher abundance of *Prevotella spp* in the luminal contents as compared to the epimural location more closely associated with the host (31).

The ruminant gastrointestinal microbiome grants many physiological and unique functions that are considered essential to maintain overall homeostasis. In general, the present study demonstrates that microbiota associated with the rumen of cattle exhibit different relative abundances of *Firmicutes* and *Bacteroidetes*. A much higher abundance of *Firmicutes* was observed in the epimural surface than the luminal contents. This result is not unexpected as the active and controlled metabolism is believed to occur at the mucosal level. An important finding of this work was that all sampled animals shared the same group of bacterial class, order and family; however, their respective abundance was significantly different between the sample locations. Marteyn et al. (32) suggested that the aerobic region within the intestines might be related to the outcome of interactions with the gut microbiota, acting as an innate immune barrier to protect the mucosal surface from anaerobic bacteria, while being recognized as a signal to promote invasion by pathogens. Perhaps this concept explains the difference in bacterial abundance in all levels between the epimural surface and the lumen samples with respect to the active *Firmicutes*. *Firmicutes* that have colonized the epimural surface may have readily available oxygen from the host essential for bacterial survival or as an advantage to growth, whereas the anaerobic environment of the lumen perhaps benefits the survival and a more balanced concentration of the *Clostridia* and *Bacilli* bacterial class. This observation could also apply to the *Bacteroidetes*, where a larger concentration of this phylum of bacteria was observed in the luminal samples as compared to the epimural surface.

In conclusion, characterizing the gastrointestinal microbiome *in vivo* is important to represent actual physiologic processes as close as possible. This study demonstrates the presence of different components and concentrations of the microbiota in two distinct location of the rumen in live cattle. This approach is crucial as many metabolically and biochemical changes in all body tissues are believed to be altered upon death (33); raising the question of how closely post-mortem samples represent normal physiologic happenings. Similar collection methods could be used in different locations of the gastrointestinal tract, allowing further investigation of the core commensal microbiome *in vivo* to study the impact of medical therapy and or environmental influences in the concentration of the metabolically-active circulating gastrointestinal bacteria in ruminants.

## Data Availability Statement

The datasets presented in this study can be found in online repositories. The names of the repository/repositories and accession number(s) can be found below: https://www.ncbi.nlm.nih.gov/, PRJNA766665.

## Ethics Statement

The animal study was reviewed and approved by IACUC at Auburn University PRN#2015-2676.

## Author Contributions

RS: designed, performed the experiment, and wrote the manuscript. EH, KH, and HH: bioinformatics and data analysis. EG: methodology development and sample collection. BN: directed the overall project and manuscript original drafting, review, and editing. PW: study's conceptualization, methodology, and surgical assistance. All authors contributed to the article and approved the submitted version.

## Funding

Funding was provided by Animal Health Research at Auburn University.

## Conflict of Interest

The authors declare that the research was conducted in the absence of any commercial or financial relationships that could be construed as a potential conflict of interest.

## Publisher's Note

All claims expressed in this article are solely those of the authors and do not necessarily represent those of their affiliated organizations, or those of the publisher, the editors and the reviewers. Any product that may be evaluated in this article, or claim that may be made by its manufacturer, is not guaranteed or endorsed by the publisher.

## Footnotes

^1^Bar Diamond® INC. #8C, Parma, ID, USA.

^2^Excenel® RTU EZ, Zoetis US Animal Health, Parsippany, NJ, USA.

^3^Meloxicam 15mg, Cipla USA, Inc., Miami, FL, USA.

^4^E.Z.N.A® Stool DNA & HiBind® matrix, Omega bio-tek®, Norcross, GA, USA.

^5^HotStarTaq Plus Master Mix Kita, Qiagen® Valencia, CA, USA.

^6^www.mrdnalab.comMR DNA, Shallowater, TX, USA.
